# Transcriptional regulatory network analysis identifies conserved cis-antisense ncRNAs in the vancomycin and ceftriaxone stress response of *Enterococcus faecalis*

**DOI:** 10.3389/fmolb.2026.1798522

**Published:** 2026-06-09

**Authors:** Javiera Pino-Gaete, Jorge Torres, Maximiliano Véliz-González, Jaime Ortega, Gladis Serrano, Gabriel Gálvez, Alex Di Genova, Álvaro Glavic, Diana Panesso-Botero, Raúl Mera-Adasme, Valentina Parra, Víctor Aliaga-Tobar, Mauricio Latorre

**Affiliations:** 1 Laboratorio de Bioingeniería; Instituto de Ciencias de La Ingeniería; Universidad de O’Higgins, Rancagua, Chile; 2 Centro de Biología de Sistemas para el Estudio de Comunidades Extremófilas de Relaves Mineros (SYSTEMIX), Universidad de O’Higgins, Rancagua, Chile; 3 Department of General Microbiology, Institute for Microbiology and Genetics, Georg-August University Göttingen, Göttingen, Germany; 4 Laboratorio de Genética Del Desarrollo, Departamento de Biología, Facultad de Ciencias, Universidad de Chile, Santiago, Chile; 5 Millennium Institute Center for Genome Regulation, Santiago, Chile; 6 Computational Biology Lab, Instituto de Ciencias de La Ingeniería, Universidad de O’Higgins, Rancagua, Chile; 7 Division of Infectious Diseases, Department of Medicine, Houston Methodist Hospital, Houston, TX, United States; 8 Center for Infectious Diseases, Houston Methodist Research Institute, Houston, TX, United States; 9 Department of Medicine, Weill Cornell Medical College, Weill Cornell Medical College, New York, NY, United States; 10 Molecular Genetics and Antimicrobial Resistance Unit (UGRA), Universidad El Bosque, Bogotá, Colombia; 11 Departamento de Química, Facultad de Ciencias, Universidad de Tarapacá, Arica, Chile; 12 Laboratory for Cell Differentiation and Metabolism, Department of Biochemistry and Molecular Biology, Facultad de Ciencias Químicas y Farmacéuticas, Universidad de Chile, Santiago, Chile; 13 Centro de Genómica y Bioinformática, Facultad de Ciencias, Ingeniería y Tecnología, Universidad Mayor, Santiago, Chile; 14 Center for Mathematical Modeling, University of Chile, Santiago, Chile; 15 Laboratorio de Bioinformática y Expresión Génica, INTA, Universidad de Chile, Santiago, Chile

**Keywords:** antibiotic stress response, ceftriaxone, *Enterococcus faecalis*, non-coding RNA, transcriptional regulatory network, vancomycin

## Abstract

**Introduction:**

*Enterococcus faecalis* survives antibiotic exposure through coordinated regulatory programs that extend beyond single resistance determinants, yet the systems-level organization of transcriptional and post-transcriptional control under clinically relevant antibiotic stress remains incompletely defined.

**Methods:**

Here, we analyzed RNA-seq profiles of *E. faecalis* V583 exposed to vancomycin or ceftriaxone and reconstructed antibiotic-activated, integrative transcriptional regulatory networks (TRNs) by projecting differentially expressed genes onto a curated TF–target template and explicitly incorporating antisense ncRNA-mRNA predicted links.

**Results:**

Both antibiotics activated a substantial shared response dominated by envelope remodeling/proteostasis and bioenergetic–nucleotide economy, while vancomycin recruited a broader condition-specific program. Despite differing scale (Van-TRN: 533 nodes/587 edges; Cef-TRN: 375 nodes/431 edges), both networks displayed sparse, hub-centered hierarchies and strong nesting, with ∼90% of ceftriaxone regulation contained within the vancomycin scaffold. From the shared ncRNA layer, we prioritized two inducible *cis*-antisense candidates, ncRNA3340 and ncRNA3683, exhibiting modular, multi-branch predicted folds, dual-site *cis* targeting, and energetics consistent with strong *cis* pairing and weaker but favorable *trans* interactions. Both ncRNAs were globally conserved across 687 clinical isolates, with low variability concentrated outside interaction-defined binding regions. Trans-target prediction points to ncRNA3340 as potentially associated with genome maintenance and cell-surface mRNAs, and ncRNA3683 with GA-like domain, glycine-alanine-rich transcripts, and a bacteriocin locus. Finally, qPCR corroborated the antibiotic-induced transcriptional changes observed for both ncRNAs.

**Conclusion:**

Together, these results provide an integrated TRN framework that connects network rewiring to conserved ncRNA-centered candidate control during vancomycin and ceftriaxone stress responses, prioritizing testable ncRNA regulons for downstream functional validation.

## Introduction

*Enterococcus faecalis* is a Gram-positive opportunistic pathogen and a leading cause of hospital-acquired infections, including endocarditis, bacteremia, and urinary tract infections. Its exceptional ability to survive antibiotic exposure relies not only on the acquisition of resistance determinants but also on complex regulatory programs that enable rapid physiological adaptation (Arias and Murray, 2012; Hollenbeck and Rice, 2012). Clinically relevant antibiotics such as vancomycin and β-lactams impose strong cell envelope stress, triggering extensive transcriptional reprogramming associated with survival, tolerance, and recovery. In enterococcal infections, this is particularly relevant for β-lactam/cephalosporin-driven envelope stress responses (including ceftriaxone exposure), which engage conserved cell-wall stress signaling systems and reshape intrinsic resistance landscapes (Kellogg et al., 2017). While individual resistance mechanisms in *E. faecalis* have been extensively studied, the global regulatory organization underlying antibiotic stress responses remains incompletely defined.

Bacterial adaptation to acute stress is inherently multilevel, involving coordinated changes in transcriptional, post-transcriptional, and post-translational regulation. Genome-wide studies across diverse bacterial systems have shown that antibiotic exposure elicits highly structured and coordinated transcriptional responses, organized within hierarchical transcriptional regulatory networks (TRNs) rather than isolated regulators. System-level TRN reconstructions have revealed global regulators, condition-specific modules, and candidate regulatory hubs that are linked to tolerance and persistence, and stress-induced evolutionary trajectories (Seshasayee et al., 2006; Fang et al., 2017; Deter et al., 2021; Zhai et al., 2023). These studies suggest a model in which antibiotic survival emerges from network-level rewiring, where partially redundant and overlapping circuits reshape metabolism, stress signaling, and cellular homeostasis.

Beyond transcription factors, non-coding RNAs (ncRNAs) have emerged as central components of bacterial regulatory networks. Small regulatory RNAs modulate mRNA stability and translation efficiency, often acting downstream of, or in parallel with, transcription factors, thereby expanding the cell’s regulatory reach (Waters and Storz, 2009; Storz et al., 2011; Nitzan et al., 2017). In *E. faecalis*, early genome-wide surveys using intergenic tiling arrays validated 11 sRNA transcripts displaying strong growth phase and stress-dependent expression patterns, including several antisense RNAs embedded in toxin–antitoxin and plasmid-associated stability modules (Shioya et al., 2011). Subsequent high-resolution mapping of primary and processed RNA ends by tagRNA-seq greatly expanded the ncRNA repertoire, revealing dozens of additional sRNAs and pervasive antisense transcription across the chromosome and mobile genetic elements, including virulence and resistance associated regions (Innocenti et al., 2015). More recently, differential RNA-seq–based transcriptome analysis has predicted approximately 150 ncRNAs in *E. faecalis*, expanding the cumulative repertoire to ∼230 candidate sRNAs and further underscoring the extensive scope of post-transcriptional regulation in this pathogen (Michaux et al., 2020). Importantly, antisense transcription has also been implicated, at least in specific contexts, in the modulation of glycopeptide resistance determinants, compatible with the plausibility of ncRNA contributions to antibiotic-related phenotypes (Torres Viera et al., 2001).

Nevertheless, only a limited subset of these ncRNAs has been functionally characterized. A few sRNAs have been experimentally linked to stress adaptation and virulence-associated phenotypes, indicating that ncRNA regulation can influence relevant physiological transitions in *E. faecalis* (Michaux et al., 2014). However, antibiotic-specific transcriptional response of ncRNAs in *E. faecalis* remain poorly resolved at the systems level, and the regulatory relationships connecting transcription factors and their shared targets under antibiotic challenge are largely unknown.

Most previous studies in *E. faecalis* have focused on differential gene expression or individual regulatory elements, providing limited insight into how transcription factors and ncRNAs collectively organize adaptive responses. Although integrative network inference approaches indicate that ncRNA-mediated regulation can be comparable to, and in some contexts rival, transcription factor control, particularly under stress conditions (Arrieta-Ortiz et al., 2020), ncRNAs remain one of the least characterized regulatory layers in *E. faecalis* during antibiotic-specific global responses. Here, we address this gap by constructing an integrated transcription factor–ncRNA regulatory network for *E. faecalis* exposed to vancomycin and ceftriaxone, leveraging RNA-seq, we inferred putative regulatory relationships, and network-based inference to resolve antibiotic-specific modules and key hubs across transcriptional and post-transcriptional layers. From this framework, we prioritize two previously uncharacterized ncRNAs responsive to both antibiotics, analyzing secondary/tertiary structural features, and infer putative regulatory relationships on selected target genes, thereby providing a systems-level view of ncRNA-centered regulation during antibiotic stress in *E. faecalis*.

## Materials and methods

### RNA-seq data source, processing, and differential expression analysis

Public RNA-seq datasets for *E*. *faecalis* V583 under vancomycin and ceftriaxone exposure were re-analyzed as described in the original studies (Jiang et al., 2022), using the V583 reference genome (NC_004668). Briefly, in the dataset used here, cultures were treated with vancomycin (256 μg/mL, 45 min) or ceftriaxone (2,048 μg/mL, 60 min) with time-matched untreated controls, and reads were retrieved from NCBI repositories (PRJNA781303 accession code). Reads were quality-checked using (FastQC) (Andrews, 2010), trimmed with Trimmomatic (Bolger et al., 2014), and aligned to NC_004668 using Bowtie2 (Langmead and Salzberg, 2012). Genomic coordinate comparison, manipulation, and annotation were performed using BEDTools (Quinlan and Hall, 2010). Strand-specific RNA-seq library orientation was evaluated using the infer_experiment.py script from the RSeQC package (Wang et al., 2012). Based on the inferred library orientation, gene-level read counts were generated with HTSeq-count (Anders et al., 2015). Differentially expressed genes (DEGs) were identified by DESeq2 (Love et al., 2014) with antibiotic versus matched-control contrasts. Statistical significance was determined using adjusted p-values (padj < 0.05) together with an absolute log2 fold change ≥1 to define differentially expressed genes. The same criteria were applied to protein-coding genes, transcription factors, and non-coding RNAs. ncRNAs were annotated by combining expressed intergenic/antisense intervals with curated *E. faecalis* ncRNA catalogs, including tiling-array validated sRNAs (Shioya et al., 2011), tagRNA-seq mapping of RNA ends (Innocenti et al., 2015), and dRNA-seq transcriptome atlases that expanded the total known set of ncRNAs (Michaux et al., 2020) and linked selected sRNAs to stress/virulence phenotypes (Michaux et al., 2014). Functional classification of antibiotic-responsive protein-coding genes was summarized by GO-class (Ensembl database) categories and visualized as Voronoi treemaps using Proteomaps software (v2.0) (Liebermeister et al., 2014).

### Reconstruction of antibiotic-activated transcriptional regulatory networks (TRNs)

Antibiotic-activated TRNs were reconstructed by integrating DEGs into a curated TF–target regulatory template for *E. faecalis*, following the same overall strategy used in the previously published EfaecalisGTN framework (Latorre et al., 2014). This model includes information on operons, transcription factors, and the connectivity between these elements for both the OG1RF and V583 strains of *E. faecalis*. For each antibiotic condition, activated networks were constructed by retaining regulatory edges that link differentially expressed genes (log2FC, significance) to their corresponding TF and ncRNA regulators, regardless of whether the regulators themselves were induced, repressed, or unchanged, and by mapping expression attributes onto both targets and regulators. *cis* ncRNA integration was performed in Cytoscape v3.10.4 (Shannon et al., 2003), by adding ncRNA→mRNA edges defined strictly by antisense genomic overlap/orientation, generating an integrated TF–ncRNA TRN per antibiotic. All data corresponding to the nodes composing the TRN are available for download in Supplementary Table S1.

### Network topology, hub inference, and functional enrichment

Network visualization and topological analyses were performed in Cytoscape v3.10.4 (Shannon et al., 2003). Global topological parameters were computed using NetworkAnalyzer (Ashkenazi et al., 2008), and hubs were prioritized with cytoHubba (Chin et al., 2014) using centrality-based rankings. Functional enrichment and term-network visualization were performed in Cytoscape using the ClueGO app (Bindea et al., 2009) with the *E. faecalis* V583 genome as background (NCBI accession NC_004668). For each antibiotic condition, the list of differentially expressed genes contained in the corresponding antibiotic-activated TRN was used as input for GO Biological Process enrichment, focusing on metabolic-process–related terms. Enrichment significance was computed using ClueGO’s hypergeometric-test framework and its default multiple-testing correction settings. Significant terms were then organized into a ClueGO term network, where nodes represent enriched GO terms and edges reflect term–term relatedness derived from shared gene membership quantified via kappa statistics, allowing ClueGO to group functionally coherent terms into modules using default network-generation parameters.

### Candidate ncRNA selection, secondary and tertiary structure modeling

Candidate ncRNAs were prioritized using combined criteria: (i) consistent antibiotic responsiveness (vancomycin and ceftriaxone), (ii) transcript support and locus boundaries from RNA-seq coverage, and (iii) network position/connectivity within the integrated TRNs. Two ncRNAs (ncRNA3340 and ncRNA3683) were selected for downstream analyses; their *cis* targets were assigned based on antisense overlap at the cognate genomic loci (details in Results section 3.4). Secondary structures of selected ncRNAs were predicted using the ViennaRNA web platform (Lorenz et al., 2011), with the standard RNAfold algorithm under thermodynamic conditions of 37 °C, 1 M monovalent salt, and the Turner 2004 energy model, extracting ensemble free energy, MFE frequency, and ensemble diversity directly from the RNAfold output (Mathews et al., 2004; Gruber et al., 2008; Lorenz et al., 2011) and finally visualized with Forna web-based tool (Kerpedjiev et al., 2015). Tertiary structures were generated using AlphaFold Server 3 in RNA mode (Abramson et al., 2024), producing five models per ncRNA, from which the model with the highest pTM score was selected without further refinement and visualized using PyMOL (The PyMOL Molecular Graphics System, Version 3.0 Schrödinger, LLC.). Putative *trans* ncRNA–mRNA interactions were predicted using IntaRNA, screening against the *E. faecalis* V583 transcriptome. Predictions were performed using default parameters, including a minimum seed length of 7 bp and explicit modeling of target site accessibility. Interaction, hybridization, and unfolding energies were retrieved from the IntaRNA v2.0 output (Mann et al., 2017).

### Conservation of ncRNAs across global clinical isolates

Homologs of ncRNA3340 and ncRNA3683 were identified across global *E*. *faecalis* clinical isolates retrieved from the PubMLST *E. faecalis* database (total *n* = 687 genomes), including isolates from Europe (*n* = 193), Asia (*n* = 163), North America (*n* = 115), Africa (*n* = 92), South America (*n* = 77), and Oceania (*n* = 47) (PubMLST) (Jolley et al., 2018). Homolog searches were performed using BLASTn (BLAST+) with a word size of 11, match reward of 1, mismatch penalty of −1, gap opening penalty of 1, and gap extension penalty of 2, retaining hits with a minimum of 80% nucleotide identity and 80% query coverage (Camacho et al., 2009). Metadata for the clinical isolate genomes included in the dendrogram analysis, including isolate identifiers, database accessions, geographic origin, and corresponding ncRNA homologs, are provided in Supplementary Table S2. Homologous sequences were identified using *E*. *faecalis* V583 (AE016830.1) as the reference. Retrieved sequences were aligned using MAFFT with default parameters, and conservation patterns were assessed from the resulting multiple sequence alignments (Katoh et al., 2019). Pairwise distances derived from the MAFFT alignments were used to reconstruct Neighbor-Joining (NJ) dendrograms, and node support was assessed by bootstrap resampling. The resulting trees were visualized and annotated according to continental origin using iTOL (Letunic and Bork, 2024).

### Prediction of *trans* targets, hybridization energetics, and motif discovery

Putative *trans* targets were predicted using IntaRNA (Busch et al., 2008) refining pairwise interactions with default parameters, which infers RNA–RNA interactions by minimizing an energy score that integrates hybridization free energy and target-site accessibility. Predictions were computed at 37 °C to approximate physiological temperature, using default seed constraints (minimum seed length 7 nt, default mismatches). Candidate targets were ranked by the minimum interaction energy and filtered using a hybridization-energy standard cutoff of ΔG ≤ −10 kcal/mol (Terai et al., 2016; Mann et al., 2017), energy-threshold filtering has been applied in IntaRNA-based interaction-ranking pipelines to enrich for more stable candidate interactions. Targeting maps and energetic components were extracted for each ncRNA–mRNA pair, including hybridization energy and unfolding energy contributions for query and target, enabling ranking and downstream interpretation. For conserved motif discovery across predicted all target regions (bindings sites), each resulting logo represents the conserved regions identified within the *cis* binding sites of each ncRNA–mRNA pair, which are also conserved across the predicted *trans* mRNA targets. Sequences were aligned (ClustalW) and then visualized as logos generated with WebLogo (Crooks et al., 2004).

### Differential expression analysis of selected ncRNAs and their target transcripts

Experimental analysis was performed using *E*. *faecalis* OG1RF (Murray et al., 1993), a widely used, genetically tractable reference strain for mechanistic studies and targeted transcriptional assays (Bourgogne et al., 2008). Cells were routinely cultured at 37 °C with agitation (150 rpm) in Medium N (Bacto Peptone 1%, yeast extract 0.5%, NaCl 0.5%, K_2_HPO_4_ 0.1%, glucose 0.1% and L-arginine 0.1%). For experiments, single colonies were used to start overnight cultures, which were refreshed into pre-warmed medium (1:20 dilution) prior to antibiotic challenge. Vancomycin and ceftriaxone concentrations were selected as the highest doses that did not alter growth after 3 h, yielding 1.25 μg/mL vancomycin and 150 μg/mL ceftriaxone. These exposure levels are within the order of magnitude of clinically achievable total plasma concentrations: for vancomycin, the selected dose falls in the sub-MIC/near-MIC range relative to *Enterococcus* susceptibility breakpoints (S ≤ 4 mg/L; EUCAST v16.0), and standard IV dosing reaches concentrations in the tens of µg/mL (e.g., ∼63 μg/mL at the end of a 1 g/60-min infusion); for ceftriaxone, a 2 g IV infusion yields peak levels of ∼257 μg/mL at the end of infusion (Pollock et al., 1982). Cultures were exposed for 3 h under the conditions above and harvested for RNA extraction. Treatment conditions for vancomycin and ceftriaxone were optimized using growth-curve analyses, selecting the highest antibiotic concentration that did not compromise bacterial viability (Supplementary Figure S1). Thus, the qPCR assays were designed as an orthogonal expression-level assessment of ncRNA/target co-variation under growth-permissive, sublethal exposure that preserves RNA integrity and enables robust detection of low-abundance regulatory transcripts, rather than as a quantitative validation of RNA-seq experiment.

Total RNA was extracted and DNase-treated following previous described protocols (Latorre et al., 2011). For strand-specific ncRNA quantification (Ho et al., 2010), ncRNA and *gyrB* (normalizer) specific cDNA was synthesized from 0.48 µg total RNA using the RevertAid First Strand cDNA Synthesis Kit (Thermo Scientific), with an ncRNA-specific primer and a *gyrB* primer (2 µL of a 10 pmol/μL primer mix) in a 20 µL reaction. mRNA quantification and qPCR assays were performed following our previously described workflow, including primer design/validation and efficiency-aware quantification (Aliaga-Tobar et al., 2026) (primer details in Supplementary Table S3). Briefly, transcript expression (ncRNA and mRNA) was analyzed in CFX Maestro, normalized to *gyrB* (EF0005), and reported as fold-change relative to untreated controls using the 2^−ΔΔCT^ method, where ΔCt_treatment = Ct_target, treatment − Ct_gyrB, treatment and ΔCt_control = Ct_target, control − Ct_gyrB, control. Amplification efficiencies were estimated from raw amplification curves using LinRegPCR (Ruijter et al., 2009). Between-group differences (treatment vs. control) were assessed using a two-tailed Student’s t-test applied to ΔCt values (n = 3 biological replicates per condition) (Rieu and Powers, 2009). For visualization, fold changes (2^−ΔΔCT^) were Log2-transformed [Log2 (^2−ΔΔCT^)] to enable symmetric representation of induction (positive) and repression (negative). Finally, to contextualize the antibiotic-induced ncRNA response against non-antibiotic stressors previously used to characterize global transcriptional remodeling in *E. faecalis*, OG1RF cultures were exposed for 3 h to sublethal, growth-permissive metal conditions: 0.5 mM CuSO_4_ or 0.5 mM FeCl_3_, selected as the doses that do not compromise viability (Latorre et al., 2014; Aliaga-Tobar et al., 2026). These conditions mirror the strategy used for antibiotic validation (non-growth-altering exposure), enabling direct comparability across perturbations. After metal exposure, total RNA was extracted, and qPCR assays and statistical analyses were conducted as described in the previous section.

## Results

### Global transcriptional and functional landscape of vancomycin- and ceftriaxone-responsive features

**FIGURE 1:**
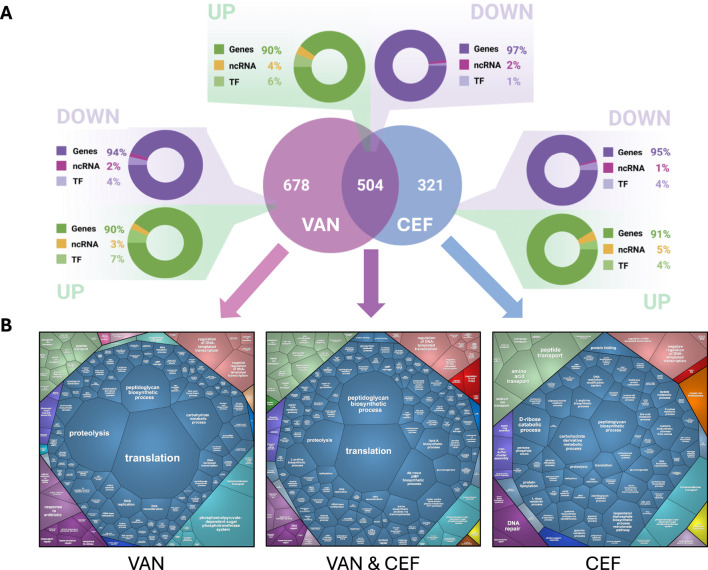
Global transcriptional and functional responses of *Enterococcus faecalis* to vancomycin and ceftriaxone. **(A)** Venn diagram summarizing differentially expressed features under vancomycin (Van) and ceftriaxone (Cef), partitioned into antibiotic-specific and shared responses. Donut charts display, for each subset and direction of change (up/down), the fraction contributed by protein-coding genes, transcription factors (TFs), and ncRNAs, normalized to the total number of differentially expressed elements in that subset. **(B)** Voronoi treemaps showing functional (GO-class) over-representation patterns of differentially expressed genes (DEGs) in Van-specific, shared (Van & Cef), and Cef-specific sets; polygon size reflects the relative contribution of each functional category within each DEG group.

To describe the antibiotic-responsive transcriptome and estimate the relative contribution of distinct regulatory layers, we compared differentially expressed protein-coding genes, transcription factors (TFs), and ncRNAs in *E. faecalis* following exposure to vancomycin or ceftriaxone (Figure 1A). Overall, vancomycin elicited the broadest condition-specific response, whereas ceftriaxone showed a more compact exclusive signature. Notably, a substantial shared component was detected across both treatments, consistent with a core stress program engaged under cell-envelope–targeting antibiotics. Under vancomycin exposure, 38% of all protein-coding genes, 44% of all transcription factor (TF) genes, and 19% of all annotated ncRNAs in the genome were differentially expressed. Under ceftriaxone exposure, the corresponding fractions were 26% (protein-coding genes), 27% (TF genes), and 12% (ncRNAs).

Across antibiotic-specific and shared subsets, the response was dominated by protein-coding genes, while TFs and ncRNAs represented a smaller fraction of the differentially expressed features. This imbalance was particularly evident for downregulated features, which were largely gene-driven, whereas the induced fraction consistently showed a relatively higher contribution from regulatory elements. Within this induced compartment, ceftriaxone displayed the strongest proportional enrichment of ncRNAs, suggesting the premise that drug-specific transcriptional activation is accompanied by measurable recruitment of post-transcriptional control. Importantly, the ncRNA component detected across all differentially expressed groups was enriched in small antisense sRNAs, consistent with pervasive antisense transcription in *E. faecalis* and with regulatory logic based on base-pairing–mediated modulation of target mRNA stability/translation rather than protein-based regulation.

To summarize the functional landscape associated with antibiotic-driven transcriptional remodeling, we grouped DEGs into vancomycin-specific, ceftriaxone-specific, and shared sets and visualized over-represented functions using Voronoi treemaps (Figure 1B). Across both antibiotics, the shared functional profile was dominated by a conserved backbone centered on translational capacity and protein homeostasis, together with prominent cell-envelope–related functions (including peptidoglycan-associated processes). This common functional architecture is consistent with convergence on envelope repair and proteostasis demands imposed by cell-wall stress, which is a hallmark of vancomycin and cephalosporin exposure in enterococci.

Beyond this core, each antibiotic displayed distinct functional weighting. Vancomycin-specific DEGs retained the shared backbone but showed an evident redistribution toward transport-centered reprogramming, including an expanded phosphoenolpyruvate-dependent PTS footprint and other membrane-associated transport functions, suggesting increased engagement of nutrient uptake/processing and envelope-linked homeostasis under vancomycin. In contrast, ceftriaxone-specific DEGs emphasized broader metabolic remodeling, with enlarged sectors linked to carbohydrate metabolism (including D-ribose catabolism), alongside a clearer representation of genome maintenance functions such as DNA repair. Collectively, these data indicate that while vancomycin and ceftriaxone converge on a shared translational/envelope remodeling axis, vancomycin uniquely strengthens transport/PTS-associated programs, previously implicated in enterococcal stress fitness, whereas ceftriaxone places relatively greater weight on metabolic diversification and DNA maintenance within its condition-specific response.

### Integrative transcriptional regulatory networks activated by vancomycin and ceftriaxone

**FIGURE 2:**
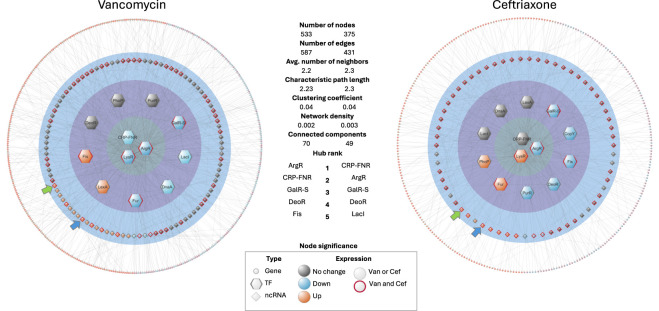
Antibiotic-specific integrative transcriptional regulatory networks (TRNs) activated by vancomycin or ceftriaxone in *Enterococcus faecalis*. Left, Van-TRN; right, Cef-TRN. Networks integrate transcription factors (TFs), ncRNAs, and antibiotic-responsive genes as nodes, with regulatory edges connecting TFs/ncRNAs to operons containing differentially expressed (DE) genes. Both models are displayed using concentric hierarchical layouts that emphasize global regulators (central layer), local regulators (intermediate layer), and peripheral ncRNA nodes with limited target connectivity. Node shape denotes entity type (gene, TF, or ncRNA), and node color indicates the expression response under the corresponding antibiotic condition (upregulated, downregulated, or unchanged). Node borders mark DE patterns across conditions: red indicates elements that are DE under both vancomycin and ceftriaxone (Van and Cef), whereas grey indicates elements that are DE in only one condition (Van or Cef). The central panel summarizes key topological properties of each TRN and lists the highest-ranking hub regulators identified by centrality analysis. Arrows highlight ncRNA3340 (green) and ncRNA3683 (blue).

We reconstructed two antibiotic-specific, integrative TRNs connecting three types of nodes (TFs, ncRNAs, and antibiotic-responsive genes) in *E. faecalis* under vancomycin or ceftriaxone exposure (Figure 2). Both networks exhibited a comparable global organization but differed in scale: the Cef-TRN contained 375 nodes and 431 edges, whereas the Van-TRN expanded to 533 nodes and 587 edges, consistent with vancomycin engaging a broader transcriptional program. Despite this size difference, both TRNs retained similarly sparse connectivity, with comparable average degree (2.2–2.3), short characteristic path lengths (∼2.2–2.3), and low clustering coefficients (0.04), indicating a hub-centered architecture rather than a uniformly connected graph.

Across both conditions, the inferred TRNs were organized into three regulatory layers. A compact set of global TFs occupied the central tier and connected to large neighborhoods of DEGs. This core was followed by a broader tier of local TFs with more restricted connectivity, and a peripheral ncRNA layer that typically linked to one or two DEG targets. These data are consistent with a hierarchical architecture in which ncRNAs are positioned to contribute to higher regulatory specificity by targeting discrete loci, often within pathway- or function-focused contexts, thereby complementing broad TF regulons with fine-grained, target-level control.

Consistent with this organization, network centrality was concentrated in a small set of hubs shared between conditions, including ArgR, CRP-FNR, GalR-S, and DeoR. The fifth-ranked hub differed between networks (Fis in Van-TRN versus LacI in Cef-TRN), suggesting subtle antibiotic-dependent redistribution of high-centrality regulators without disrupting the overall architecture. Comparative mapping further revealed strong nesting between conditions, with ∼90% of the Cef-TRN content contained within the Van-TRN. This extensive proportion of shared nodes and edges between the Cef-TRN and the Van-TRN, with the Cef-TRN largely embedded within the Van-TRN, indicates that ceftriaxone primarily activates a core regulatory scaffold that is also engaged under vancomycin. In contrast, vancomycin recruits additional condition-specific nodes and connections beyond this shared backbone, consistent with a broader activation of envelope stress–associated transcriptional control.

### Metabolic processes overrepresented in antibiotic-activated regulatory networks

**FIGURE 3:**
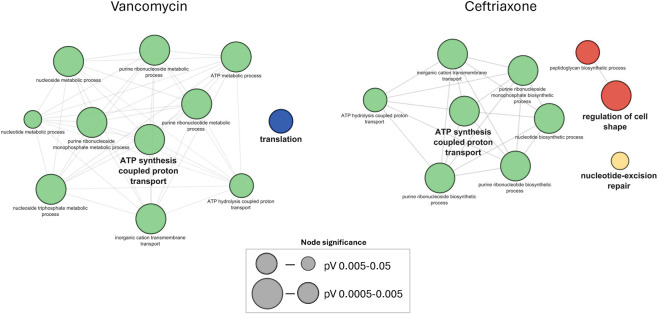
Metabolic-process enrichment networks derived from antibiotic-activated TRNs. Differentially expressed genes from the vancomycin-activated (left) and ceftriaxone-activated (right) TRNs were subjected to Gene Ontology (GO) Biological Process enrichment and visualized as ClueGO term networks focused on metabolic processes. Nodes represent significantly enriched GO terms, and edges denote term–term relatedness based on shared gene membership (see Methods). Node size scales with enrichment significance (p-value bins shown in the legend). Node colors indicate the functional groups (clusters) whose names are highlighted in bold.

Mapping DEGs from the antibiotic-activated TRNs onto ClueGO-metabolic process enrichment networks revealed a conserved core response shared by vancomycin and ceftriaxone, together with antibiotic-specific metabolic signatures (Figure 3). In both conditions, the most central and highly connected module was ATP synthesis coupled proton transport, surrounded by tightly linked terms related to nucleotide (purine) metabolism and salvage. This shared architecture indicates that, across antibiotics, the dominant enriched signal concentrates on bioenergetic/homeostatic processes and nucleotide economy, rather than on isolated pathways. Notably, transcriptomic studies of *E. faecalis* under stress conditions frequently report concurrent remodeling of nucleotide metabolism and strong regulation of ATP synthase components, consistent with the prominence and connectivity of these modules in the inferred networks.

Despite this shared core, the vancomycin network displayed an additional, clearly separated enrichment centered node on translation process. These features define a vancomycin-specific functional profile that is distinguished by enrichment in RNA-level processes and protein synthesis/processing, alongside the conserved energy/nucleotide backbone. This pattern aligns with prior genome-wide analyses of the *E. faecalis* cell wall–targeting antibiotic stimulon, where vancomycin exposure is associated with broad physiological reallocation including repression/rebalancing of translation and energy metabolism alongside envelope-associated programs (Abranches et al., 2014), and with the stringent-response framework in *E. faecalis* that couples translation capacity with nucleotide/energy homeostasis (Gaca et al., 2012). In addition, for vancomycin exposure, lipid II binding is predicted to enrich for downstream programs that is consistent with envelope repair/remodeling and proteostasis, as reflected by the strong envelope-centered backbone observed in the Van-TRN (Stogios and Savchenko, 2020).

For its part, the ceftriaxone network retained the same energy/nucleotide core but showed stronger enrichment around cell shape regulation (linked to peptidoglycan-related terms) and a distinct DNA-centric neighborhood including nucleotide-excision repair and DNA topological change. The association between ceftriaxone exposure and the enrichment of cell-wall–related functional terms is consistent with the known β-lactam mode of action, which can inhibit PBPs and may lead to downstream envelope stress and, in some contexts, autolysis-related effects. Finally, ceftriaxone uniquely displays a discrete DNA-centric node dominated by nucleotide-excision repair, which may reflect engagement of genome maintenance programs under antibiotic-associated stress conditions, without implying direct DNA targeting by the drug (Žgur-Bertok, 2013).

This functional coupling is also consistent with the resistance architecture expected for *enterococci* under glycopeptide versus cephalosporin challenge. The prominence of cell-shape/peptidoglycan-linked terms is consistent with β-lactam PBP inhibition, but in enterococci this stress is additionally routed through intrinsic cephalosporin resistance determinants that depend on low-affinity PBPs and envelope stress signaling (including CroRS and related regulatory modules) (García-Solache and Rice, 2019). Thus, the comparatively “nested” Cef-TRN can be interpreted as activation of a shared envelope-stress scaffold, while drug-specific differences reflect the distinct points of interference (lipid II binding versus PBP inactivation) and the regulatory systems that modulate cephalosporin outcomes in *E. faecalis*.

### Selection and structural profiling of antibiotic-inducible antisense ncRNAs

Candidate ncRNAs were prioritized from the subset of ncRNAs that were differentially expressed under both vancomycin and ceftriaxone (shared ncRNA layer; see Supplementary Table S1) using the following criteria: (i) consistent induction across conditions, (ii) clear RNA-seq transcript support with well-defined locus boundaries in the *E. faecalis* V583 reference genome, and (iii) an unambiguous *cis*-antisense architecture with overlap to a single protein-coding locus outside an operon context, enabling direct assignment of the cognate *cis* target.

From the antibiotic-responsive ncRNA layer shared by both TRNs, we selected ncRNA3340 and ncRNA3683 because they best satisfied our selection criteria and showed the most favorable overall parameters. Both were prioritized as candidate post-transcriptional regulatory elements consistently induced under vancomycin and ceftriaxone treatment. Both are annotated as cis-antisense sRNAs, transcribed opposite to their cognate loci and predicted to base-pair with overlapping mRNAs and potentially influence gene expression through base pairing with overlapping mRNAs. Consistent with this regulatory mode, each ncRNA was associated with a single primary *cis* target outside an operon context (Latorre et al., 2014), consistent with a highly specific candidate architecture centered on one antibiotic-responsive gene per ncRNA. All coordinates refer to the *E*. *faecalis* V583 reference genome (AE016830.1). In this genome, ncRNA3340 is a 708-nt transcript (37.4% GC) located at 1,198,329–1,199,037 and overlapping EF1231 (1,198,352–1,199,176) in antisense orientation, whereas ncRNA3683 is an 823-nt transcript (39.6% GC) located at 1,336,636–1,337,459 and overlapping EF1362 (1,336,711–1,337,478) in antisense orientation (full information and the sequences of both ncRNAs have been added to Supplementary Table S4).

**FIGURE 4:**
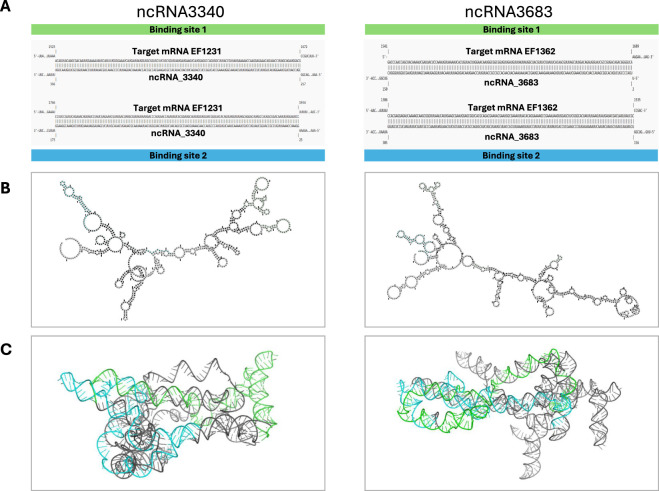
Selection and structural modeling of antibiotic-inducible antisense ncRNAs and their predicted *cis* interactions. **(A)** Predicted ncRNA–mRNA interaction maps for ncRNA3340–EF1231 and ncRNA3683–EF1362, highlighting two binding sites per ncRNA (binding site 1 and 2) and the corresponding paired regions in the target mRNAs. **(B)** Predicted secondary structures for ncRNA3340 and ncRNA3683, with interaction-associated domains highlighted (colors correspond to the two binding regions). **(C)** Predicted tertiary fold visualizations for each ncRNA derived from AlphaFold Server 3 modeling, shown to illustrate overall RNA architecture and spatial separation of the interaction-associated domains (see Supplementary Table S4 for modeling metrics and interaction energetics).

Targeting maps predicted two binding sites for each ncRNA–mRNA pair (Figure 4A; Supplementary Table S5). More specifically, IntaRNA identified two predicted interaction segments for each pair: for ncRNA3340, ncRNA regions 217–366 and 25–173 paired with target-sequence regions 1,523–1,672 and 1,716–1,864, respectively; for ncRNA3683, ncRNA regions 156–305 and 2–150 paired with target-sequence regions 1,386–1,535 and 1,541–1,690, respectively. These predicted sites are consistent with interaction within the overlapping cis-antisense genomic context of each ncRNA–target pair. ncRNA3340 was associated to EF1231, encoding a metallophosphoesterase, a functional class repeatedly implicated in Gram-positive envelope adaptation and kinase–phosphatase circuits modulating cephalosporin susceptibility in *E. faecalis* (Kristich et al., 2011), as well as in broader cell-wall antibiotic responses enriched in regulatory and phospho-signaling function (Darnell et al., 2019). Both sites showed strongly favorable interaction energies (−243.8 and −230.2 kcal/mol) across two distinct mRNA segments paired by separated ncRNA regions, compatible with a modular targeting configuration rather than reliance on a single seed. ncRNA3683 was linked to EF1362 (lipoprotein), consistent with surface lipoproteins contributing to *E. faecalis* virulence and stress fitness (Reffuveille et al., 2011; Reffuveille et al., 2012). Two sites were also detected with strong interaction/hybridization energies (∼−233 to −246 and ∼−290 kcal/mol), but with unfolding penalties (23–29 kcal/mol), suggesting that duplex formation requires local structural opening.

The predicted folds of ncRNA3340 and ncRNA3683 are compatible with modular, multi-branch architectures in which two peripheral stem–loop domains (cyan/green in Figures 4B,C) extend from a compact core, creating apical loops and internal bulges that could provide accessible interaction regions for antisense pairing. This organization offers a plausible structural context for the two interaction segments mapped for each ncRNA against its *cis* target, because independent loop-rich regions can support parallel or alternative pairing routes rather than a single contiguous “seed”. This aligns with the general principle that bacterial sRNA–mRNA recognition often relies on short, localized pairing domains that drive specificity while the rest of the RNA serves as a structural scaffold.

Consistently, RNAfold ensemble metrics (MFE frequency = 0%, high ensemble diversity) indicate that both ncRNAs likely populate multiple near-isoenergetic conformers, which would modulate loop accessibility and facilitate engagement of alternative binding segments under stress. Structured regulatory RNAs frequently use hairpin apical loops as interaction platforms that enable base pairing and conformational switching, compatible with this interpretation. Although the low-confidence 3D scores (best-model pTM = 0.11) preclude atomic-level inference. Accordingly, the AlphaFold models are included only as illustrative qualitative fold visualizations and not as high-confidence structural evidence; their value here is to provide a conceptual structural context for the accessibility-aware interaction framework used by IntaRNA, which explicitly incorporates local RNA conformational openness in target prediction, suggesting that the two interaction-associated domains occupy spatially separable surfaces that could enable redundant or condition-dependent antisense engagement. Together, modular stem–loops, candidate toeholds, and ensemble heterogeneity reinforce prioritization of ncRNA3340 and ncRNA3683 as *cis*-antisense regulators with dual-site targeting potential in the shared antibiotic response.

### Global conservation of antibiotic-inducible ncRNAs across *Enterococcus faecalis* clinical isolates

**FIGURE 5:**
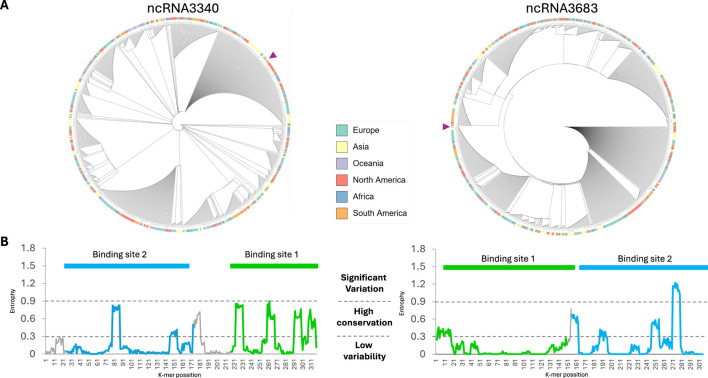
Conservation of ncRNA3340 and ncRNA3683 across global *Enterococcus faecalis* clinical isolates. **(A)** Circular dendrograms built from ncRNA sequence alignments showing the global distribution of ncRNA3340 (left) and ncRNA3683 (right) across isolates from different continents (outer ring color code). Purple markers indicate the reference isolates analyzed in this study. **(B)** Per-position sequence variability plotted as entropy along each ncRNA. Colored bars denote the two predicted binding regions (binding sites 1 and 2). Dashed lines indicate low-variability and high-variation thresholds used to highlight conserved versus variable segments.

To determine whether the two antibiotic-inducible ncRNAs (ncRNA3340 and ncRNA3683) are restricted to specific hospital lineages or broadly distributed across *E. faecalis*, we assessed their conservation across a geographically diverse collection of clinical isolates (Figure 5A). For both candidates, the circular dendrograms is consistent with widespread conservation, with isolates from Europe, Asia, Oceania, North America, South America, and Africa interspersed throughout the trees rather than segregating into continent-specific clades. In both cases, the isolates analyzed in this study (purple marker) clustered within mixed-origin groups, indicating that the ncRNA sequences carried by the strain under study are representative of the global diversity of hospital-associated *E. faecalis* rather than phylogeographic outliers. Overall, the dendrogram topology is consistent with high sequence conservation and the absence of obvious geographic structure for either ncRNA across worldwide clinical backgrounds.

To quantify sequence constraint at nucleotide resolution, we calculated the frequency of nucleotide variation across all homologous sequences for each ncRNA and summarized it as a per-position entropy profile (Figure 5B; Supplementary Figure S2). In both ncRNA3340 and ncRNA3683, overall variability was low and concentrated on a limited number of localized peaks. Importantly, the two predicted binding sites involved in *cis* interaction with their target mRNAs exhibited consistently reduced entropy, indicating that these interaction interfaces are among the most conserved regions of each ncRNA. This pattern suggests strong purifying selection on the predicted pairing domains, consistent with the notion that functional base-pairing regions in regulatory bacterial RNAs tend to be conserved to maintain target recognition across strains. Collectively, the broad phylogenetic distribution and the low variability within binding interfaces suggest that ncRNA3340 and ncRNA3683 represent widely shared antisense regulatory elements across hospital lineages, reinforcing their potential relevance as conserved components of the antibiotic-responsive transcriptional program in *E. faecalis*.

### Trans-regulatory expansion of antibiotic-induced ncRNAs

**FIGURE 6:**
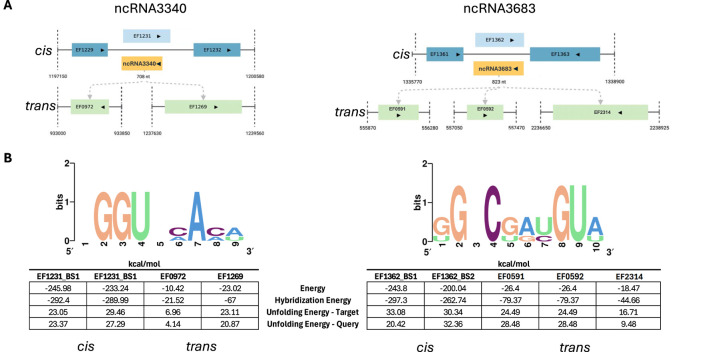
Predicted *trans*-target landscape, conserved interaction motifs, and energetics for ncRNA3340 and ncRNA3683. **(A)** Genomic context of *cis* and predicted *trans* targets for ncRNA3340 (left) and ncRNA3683 (right). **(B)** Sequence logos summarizing conserved positions within the predicted mRNA interaction segments across *cis* and *trans* targets for each ncRNA, together with predicted interaction/hybridization energies and unfolding penalties (target and query).

To extend the predicted regulatory impact suggesting the broader post-transcriptional reach of the two antibiotic-induced ncRNAs, we performed an additional bioinformatics search using the IntaRNA tool, which allows us to predict candidate *trans* targets for ncRNA. (Figure 6A). Candidate *trans* targets were identified with IntaRNA by screening the complete annotated CDS set of the *E. faecalis* V583 genome, and the genes discussed here correspond to the predicted coding-sequence hits recovered in that analysis. No additional filtering by expression or differential expression was applied at this stage; therefore, these loci are presented as computationally predicted candidate *trans* targets. For ncRNA3340, in addition to its *cis* association with EF1231, targeting analyses using IntaRNA predicted two *trans* targets: EF0972 (DNA repair exonuclease family protein) and EF1269 (cell wall surface anchor family protein). These candidates suggest that ncRNA3340 may sit at the intersection element of genome maintenance and cell-envelope/host-interface functions, processes repeatedly implicated in antibiotic-stress survival and infection-associated adaptation. DNA repair pathways may influence survival during antibiotic stress and could contribute to resistance emergence via stress-linked mutagenesis (Žgur-Bertok, 2013; Giroux et al., 2017), while LPxTG/surface-anchored determinants contribute to adhesion/biofilm phenotypes linked to persistence and treatment refractoriness (Chuang et al., 2009; Hendrickx et al., 2009).

For ncRNA3683, beyond its cis-antisense relationship with EF1362, we identified three candidate *trans* targets: EF0591 and EF0592 (glycine-alanine-rich like (GA-like) domain–containing proteins, partial) and EF2314 (putative bacteriocin). The adjacency and shared annotation of EF0591/EF0592 suggest that they may represent a single disrupted ORF or pseudogene remnant split by frameshift or indels, consistent with GA-like domains, defined here as glycine–alanine–rich, low-complexity regions commonly found in Gram-positive cell-surface proteins involved in envelope structure and host or environmental interactions (Johansson et al., 1995; Johansson et al., 1997). Bacteriocin loci are frequently linked to within-host competition, colonization success, and virulence-associated traits, and can be embedded in broader stress-response programs (Graham et al., 2017).

Energetic profiling suggests that both ncRNAs share a similar quantitative signature: predicted ncRNA–mRNA interactions tend to have favorable hybridization energies, while the effective interaction scores are constrained by target-site accessibility (Figure 6B). Consistent with their cis-antisense annotation, *cis* pairs show markedly stronger predicted binding than *trans* candidates, reflecting their longer stretches of complementarity and lower minimum free energy. In contrast, the predicted *trans* interactions are weaker overall and are associated with shorter aligned segments, which is consistent with the energy and alignment-length distributions shown. Accordingly, the conserved 4-nt cores in the interaction alignments may represent minimal reusable recognition motifs, these conserved cores were identified directly from the predicted ncRNA–mRNA interaction alignments shown in Figure 6B. For ncRNA3340, the shared pattern corresponded to a GGUxxA motif, whereas for ncRNA3683 the aligned interactions retained a GxCxxxGU pattern, consistent with the presence of short conserved recognition signatures across the predicted *trans* interactions that may be compatible with broader candidate *trans*-targeting without requiring long sequence identity.

Despite this common framework, the ncRNAs differ in *trans*-target behavior. ncRNA3340 shows a more heterogeneous *trans* landscape, suggesting stronger dependence on local RNA structure and context-specific accessibility. In contrast, ncRNA3683 displays a more coherent *trans* signature: EF0591 and EF0592 have nearly identical energetics, suggesting a split/pseudogenes locus and/or recognition of a repeated conserved motif across related transcripts. The weaker pairing predicted for EF2314 suggests a more conditional, context-dependent interaction rather than a dominant *trans* target. Overall, both ncRNAs follow a *cis*-biased pairing logic, while ncRNA3683 appears to operate through a more motif-consistent *trans* program than ncRNA3340, implying differences in robustness and selectivity of their post-transcriptional regulons.

### Validation of antibiotic-induced ncRNA changes and predicted targets under vancomycin and ceftriaxone exposure

**FIGURE 7:**
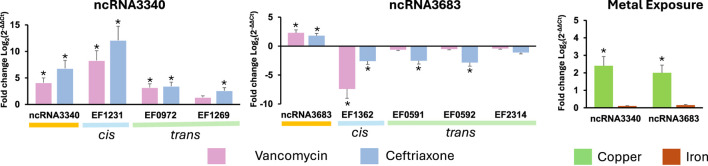
Antibiotic- and metal-dependent expression changes of ncRNA3340 and ncRNA3683 and their predicted cis/trans target sets. qPCR fold-change for ncRNA3340, ncRNA3683, and selected predicted cis/trans targets under vancomycin and ceftriaxone, and under metal exposure (0.5 mM CuSO_4_ or 0.5 mM FeCl_3_; 3 h). Bars indicate mean ± SD of the fold-change Log_2_ (2^−ΔΔCT^) across three biological replicates (n = 3); asterisks denote significant differences relative to the corresponding control (two-tailed Student’s t-test on ΔCt values). To improve resolution of expression differences, fold-changes (2^−ΔΔCT^) are displayed on a Log2, enabling symmetric visualization of induction (positive values) and repression (negative values).

To assess whether the ncRNA–mRNA predicted links inferred from the integrative TRNs were directionally consistent with the global expression patterns observed in *E. faecalis* OG1RF, we used qPCR assays to quantify antibiotic-dependent expression changes in each ncRNA together with its cis target and predicted trans candidates (Figure 7). This provides an expression-based context to interpret the interaction energetics reported previously.

Within the ncRNA3340 module, ncRNA3340 was induced under both antibiotics, and its *cis* target EF1231 showed the largest positive shift. The predicted *trans* target EF0972 also increased under both treatments, consistent with a shared induction profile, whereas EF1269 displayed a ceftriaxone-biased response, increasing mainly under ceftriaxone and only marginally under vancomycin. Thus, ncRNA3340 is associated with a conserved branch (EF1231/EF0972) and a ceftriaxone-skewed component (EF1269).

For ncRNA3683, induction was also observed under both antibiotics, but its targets followed a distinct pattern. The *cis* target EF1362 showed greater repression under vancomycin than under ceftriaxone, yielding an inverse ncRNA–mRNA relationship compatible with, but not demonstrative of, with negative *cis* regulation. The *trans* candidates EF0591 and EF0592 showed highly similar repression patterns (again strongest under ceftriaxone), with the largest decrease under ceftriaxone, whereas EF2314 remained close to basal levels.

Integrating these profiles with interaction energetics (Figure 6B) offers an expression-level pattern that is compatible with the predicted ranking: *cis* interactions, predicted to be most favorable, align with the largest absolute expression changes (EF1231 induction; EF1362 repression), whereas weaker *trans* interactions tend to associate with smaller shifts or no detectable change. This is particularly apparent for ncRNA3683, where GA-like targets show measurable repression while EF2314 (least favorable predicted pairing) remains largely unchanged, consistent with prioritization of these pairs for downstream validation.

To assess whether the induction of ncRNA3340 and ncRNA3683 reflects an antibiotic-specific signature or a broader stress-responsive program, we challenged *E. faecalis* OG1RF with two orthogonal metal stressors (copper and iron) that have been extensively used to characterize genome-wide transcriptional remodeling in *E. faecalis* under non-lethal metal excess (Abrantes et al., 2011; Latorre et al., 2014; Latorre et al., 2018; Ali et al., 2022). We applied 0.5 mM CuSO_4_ or 0.5 mM FeCl_3_ for 3 h, matching the non-growth-altering rationale used for our antibiotic validation. Copper and iron are biologically relevant stresses for *enterococci* in host and environmental contexts, and trigger distinct homeostatic programs: copper excess elicits broad transcriptome rewiring beyond the *cop* operon, including detoxification, membrane-associated functions, and metabolic adjustments, whereas iron excess activates a partially oxidative-stress–mediated response centered on metal transport and redox control (López et al., 2012). Conceptually, this copper/iron contrast provides a graded stress contrast: copper typically imposes a higher acute intracellular burden due to redox cycling, protein mismetallation, and thiol stress, while iron responses can be more buffered and condition-dependent. Under these comparable conditions, both ncRNA3340 and ncRNA3683 increased in abundance during copper exposure, whereas iron did not measurably alter their levels relative to basal controls (Figure 7). Importantly, we do not interpret this experiment as evidence that copper or iron reproduces the primary mechanism of vancomycin or ceftriaxone, nor as direct proof of cell-wall damage; rather, both metals were used as orthogonal, non-antibiotic perturbations to test response specificity. These results indicate that induction of both ncRNAs is not a generic response to metal exposure and instead is preferentially associated with copper stress, compatible with the interpretation that their activation occurs under a subset of more disruptive perturbations that also include vancomycin and ceftriaxone.

## Discussion

Vancomycin and ceftriaxone elicited a largely shared, cell-envelope–centered stress program in *E. faecalis*, but with different depth and wiring, consistent with their distinct primary targets and enterococcal physiology. Across the transcriptome, vancomycin recruited a broader condition-specific shift than ceftriaxone, while a substantial common core was co-activated by both drugs. This core and drug-specific organization agrees with systems-level views in which antibiotic survival emerges from coordinated network rewiring rather than single genes (Fang et al., 2017; Sastry et al., 2019; Arrieta-Ortiz et al., 2020; Yang et al., 2024; Zhuang et al., 2024). Induced compartments showed a relatively larger contribution of regulatory features (TFs and ncRNAs) than repressed compartments, suggesting that activation under drug stress preferentially engages regulatory layers, whereas repression reflects downstream economization of protein-coding functions (Voglauer et al., 2025).

Functional profiling reinforced this interpretation: both antibiotics converged on a backbone dominated by protein homeostasis and envelope remodeling (translation/proteolysis and peptidoglycan-related functions), consistent with cell-wall–active stressors. Comparable coupling between envelope perturbation, proteostasis, and energetic rebalancing has been described across bacteria, including contexts where ATP homeostasis is a determinant of stress tolerance (Pichon and Felden, 2005; Voglauer et al., 2025). In our dataset, vancomycin uniquely expanded signatures linked to antibiotic response and PTS-associated carbohydrate import/processing, consistent with glycopeptide stress promoting broader physiological reallocation beyond the conserved core (Voglauer et al., 2025). Ceftriaxone, in contrast, displayed comparatively stronger signals in carbohydrate pathway remodeling and DNA repair/maintenance terms, aligning with evidence from other systems that β-lactam–linked envelope stress can engage genome maintenance and metabolic diversification (Wang et al., 2023).

At the regulatory-network level, both antibiotic-specific TRNs showed a conserved hierarchical, hub-centered design with sparse connectivity, consistent with general bacterial TRN principles where global regulators coordinate broad neighborhoods and local regulators refine outputs (Fang et al., 2017; Sastry et al., 2019).

In particular, the conserved presence of regulators acting as hubs within the network, is consistent with global regulators that connect antibiotic-induced envelope stress to core metabolic reallocation. In *E. faecalis*, ArgR/AhrC-family control links arginine utilization with ATP/pH homeostasis and biofilm-associated infection phenotypes (Frank et al., 2013); importantly, growth in arginine can decrease susceptibility to β-lactams including ampicillin and ceftriaxone (Snell et al., 2024), providing a plausible route for enhanced ArgR connectivity under ceftriaxone stress. CRP/FNR-like regulation is represented by Ers, implicated in oxidative-stress survival and intracellular persistence (Riboulet-Bisson et al., 2008; Riboulet-Bisson et al., 2009). In parallel, LacI/GalR-type carbohydrate regulators implement catabolite repression in *E. faecalis* (e.g., MalR (Grand et al., 2020)), compatible with the idea that GalR–S-like nodes help route sugar-phosphate resources toward envelope repair during cell-wall challenge (Abranches et al., 2014). Finally, DeoR-family regulation links deoxyribonucleoside catabolism to nucleotide economy (Škerlová et al., 2014), which is tightly connected to stringent-control programs in *E. faecalis* (Gaca et al., 2012) and may explain why nucleotide-centered modules remain core features of both antibiotic-activated TRNs.

In addition, an important result was the strong nesting of ceftriaxone regulation within vancomycin regulation (∼90% of the Cef-TRN contained in the Van-TRN), consistent with a model in which ceftriaxone activates a subset of a shared scaffold, whereas vancomycin additionally recruits extended regulatory structure (Jiang et al., 2025). This is conceptually consistent with vancomycin’s direct blockade of peptidoglycan precursor utilization driving a strong envelope stress response, while ceftriaxone evokes a more constrained program that still overlaps the same core circuitry.

A key distinction is that vancomycin directly targets the peptidoglycan precursor rather than a protein enzyme, and resistance can emerge by remodeling the precursor itself. Vancomycin binds lipid II through an extensive hydrogen-bond network to the D-Ala–D-Ala terminus, and high-level resistance is commonly achieved by replacing this motif with D-Ala–D-Lac (or, in other operon types, D-Ala–D-Ser), which markedly reduces drug affinity (Stogios and Savchenko, 2020). In enterococci, these changes are mediated by *van* operons (e.g., vanA/vanB) that encode a coordinated enzymatic cascade under two-component control (VanR/VanS), redirecting precursor biosynthesis and eliminating susceptible D-Ala–D-Ala substrates (García-Solache and Rice, 2019). Importantly, the canonical VanB determinant was originally described in *E. faecalis* V583 (Evers et al., 1993), providing a relevant resistance backdrop for interpreting vancomycin-triggered network expansion. Within this framework, the broader depth of the Van-TRN is consistent with the need to coordinate envelope remodeling with global homeostasis (proteostasis/translation and energy–nucleotide economy) under direct precursor blockade and precursor-rewiring resistance logic.

For ceftriaxone, the observed TRN structure is compatible with the well-established intrinsic cephalosporin resistance of enterococci and its regulatory control. Clinical enterococci exhibit an expected resistance phenotype to cephalosporins, and in *E. faecalis* β-lactam/cephalosporin outcomes are shaped by low-affinity PBPs (notably PBP4) together with envelope stress signaling and regulatory loci required for cephalosporin resistance (García-Solache and Rice, 2019). Recent work has also shown that structural/regulatory changes in PBP4 can shift β-lactam susceptibility in *E. faecalis* (Rice et al., 2018). In this context, the stronger weighting of cell-shape/peptidoglycan-linked terms in the ceftriaxone enrichment landscape and the tight embedding of ceftriaxone regulation within the vancomycin scaffold can be interpreted as deployment of a conserved envelope-stress core with a ceftriaxone-specific “PBP-filtered” branch. This offers a possible explanation for why ceftriaxone primarily activates the shared backbone captured by the integrative TRN while still producing distinct signatures in cell-wall functional organization and associated regulatory wiring.

A central contribution of this study is the explicit placement of ncRNAs within the TRN hierarchy. ncRNAs occupy a peripheral yet specificity-enhancing layer, typically linked to one or two targets, suggesting potential high-resolution regulatory nodes within a TF-driven scaffold. This is consistent with sRNA paradigms, where short pairing domains (seed/toeholds) may contribute to specificity and accessibility gates interaction initiation in bacteria and also multicellular eukaryotes (Waters and Storz, 2009; Papenfort et al., 2010; Jiang et al., 2025). Global interaction-mapping studies in other bacteria show that stress-responsive sRNAs can reorganize condition-specific regulons with precision comparable to TF control (Han et al., 2016), consistent with the interpretation that the ncRNA layer captured here is functionally meaningful (Bombelli et al., 2026).

Within this framework, ncRNA3340 and ncRNA3683 emerged as robust elements of the shared antibiotic response: both are induced by vancomycin and ceftriaxone, are predicted *cis*-antisense sRNAs, and display dual-site interaction potential with their cognate *cis* prominent targets. In addition, ncRNA3340 and ncRNA3683 were not assigned to any of the hub transcription factors (ArgR, CRP–FNR, GalR–S, DeoR) nor to other transcription factors in our curated TF–target framework. This lack of TF connectivity suggests that their regulation is largely decoupled from the dominant hub-driven programs, operating through independent control routes. Consequently, these two ncRNAs likely contribute an additional, more specific regulatory layer that complements global TF-mediated rewiring during antibiotic stress.

The added metal controls are compatible with a stress-selective interpretation of these ncRNAs. When tested under comparable, growth-permissive non-lethal exposures, both ncRNA3340 and ncRNA3683 were induced by copper but not by iron. Given that copper imposes a comparatively higher cellular burden, through redox cycling, thiol/protein disruption, and broad perturbation of envelope and metabolic homeostasis, whereas iron-triggered responses are more contingent on speciation, intracellular buffering, and tightly regulated uptake/storage, this pattern is consistent with these ncRNAs being preferentially engaged under high-intensity stress conditions rather than representing a single antibiotic-specific defense mechanism. This interpretation is further reinforced by their shared induction under two antibiotics with distinct modes of action, vancomycin and ceftriaxone.

Further, their predicted targets are consistent with antibiotic-relevant physiology. For ncRNA3340, EF1231 encodes a metallophosphoesterase-annotated function, consistent with the importance of kinase–phosphatase signaling in Gram-positive envelope adaptation and intrinsic cephalosporin susceptibility control in *E. faecalis* (IreK–IreP) (Kristich et al., 2011; Han et al., 2016; Han et al., 2024). For ncRNA3683, the *cis* target EF1362 encodes a predicted lipoprotein, a class repeatedly associated with surface fitness, stress tolerance, and virulence traits in *E. faecalis* and related pathogens (Reffuveille et al., 2011; Reffuveille et al., 2012). Directionality differed between modules: ncRNA3340 and EF1231 were co-induced, compatible with shared upstream control or non-canonical antisense outcomes, whereas ncRNA3683 induction coincided with EF1362 repression, more consistent with negative regulation in *cis*. Such heterogeneity is compatible with broader antisense/sRNA literature in which pairing can promote decay or, depending on RNase access and context, stabilize transcripts or alter translation without simple mRNA decreases (Liang et al., 2011; Han et al., 2024).

A plausible connection to the target space is that both ncRNA modules map to functions commonly engaged during high-burden stress. ncRNA3340 is antisense to EF1231 (metallophosphoesterase annotation) and is linked to candidate targets including genome-maintenance and surface-associated loci, whereas ncRNA3683 is antisense to EF1362 (predicted lipoprotein) and is linked to surface/GA-like and bacteriocin-associated candidates. Under severe stressors, such as cell-wall–active antibiotics and copper, modulating envelope-associated and homeostatic functions could be repeatedly recruited, whereas the lack of induction under iron suggests that not all perturbations trigger the same post-transcriptional layer. Importantly, this remains hypothesis-generating and does not establish causality between each ncRNA and individual targets, which will require downstream functional testing.

Co-induction does not conflict with a cis-antisense architecture, because antisense pairing does not necessarily yield an inverse mRNA abundance pattern. *cis*-antisense RNAs can modulate gene output primarily at the level of translation and/or RNA processing, and in some contexts can even increase target stability by protecting cleavage sites, so both transcripts may rise when the locus is transcriptionally activated under stress (Georg and Hess, 2011; Stazic et al., 2011). More generally, base-pairing RNAs can remodel mRNA structure and shift stability/translation without requiring a decrease in total mRNA levels (Stazic et al., 2011). Accordingly, we interpret the ncRNA3340–EF1231 co-induction as compatible with shared upstream activation plus post-transcriptional tuning rather than as evidence against antisense regulation.

Structural and energetic profiling suggests a shared interaction model: both ncRNAs show modular, multi-branch architecture with loop/bulge-rich domains that may provide accessible pairing regions, and both populate heterogeneous secondary-structure ensembles, implying that accessibility and conformational switching modulate which pairing interface dominates. This interpretation aligns with sRNA systems where interactions may initiate at exposed loops and extend through duplex extension, with engagement shaped by local unfolding costs and target accessibility (Waters and Storz, 2009; Nakatsu et al., 2019). Although 3D models should be treated qualitatively, they still offer qualitative context for multi-site targeting by suggesting separable interaction surfaces.

Two additional observations are consistent with broad clinical relevance. Both ncRNAs are globally conserved across geographically diverse hospital isolates without a strong phylogeographic signal, consistent with classic observations of shared core features across enterococcal lineages (Gottesman and Storz, 2011). Moreover, low variability within interaction-defined binding-site regions suggests purifying selection on recognition determinants, paralleling patterns reported for functional sRNAs in other pathogens (Pichon and Felden, 2005; Acuña et al., 2021).

Finally, integrating *trans*-target prediction with energetics and qPCR validations yields a coherent, hypothesis-generating framework for regulon breadth. For both ncRNAs, energetics separates strong *cis* pairing from weaker but favorable *trans* pairing, implying that *trans* regulation may depend on short nucleation motifs and accessibility (Pichon and Felden, 2005). The near-identical energetic and expression profiles of EF0591 and EF0592 suggest a split/pseudogenes GA-like locus and suggest ncRNA3683 may recognize a motif repeated across closely related transcript fragments, whereas ncRNA3340 displays a more heterogeneous *trans* landscape, potentially making its *trans* effects more conditional. Such conditionality is common across bacteria, where pairing strength alone does not guarantee regulatory output and can depend on growth phase and RNA-processing context, as shown by global interaction-mapping approaches (Han et al., 2016). Overall, our results are consistent with a model in which vancomycin and ceftriaxone activate a shared hierarchical TRN scaffold in *E. faecalis*, within which conserved *cis*-antisense ncRNAs represent specificity-enhancing candidate control points that diversify post-transcriptional outputs and may contribute to antibiotic-adaptive physiology in ways that are consistent with principles established in other pathogens.

## Conclusion

In this study, we provide a systems-level view of antibiotic-responsive regulation in *E. faecalis* by integrating RNA-seq differential expression with curated regulatory interactions to reconstruct antibiotic-specific TRNs under vancomycin and ceftriaxone exposure. Both antibiotics activated a substantial shared core program dominated by cell-envelope remodeling and proteostasis, whereas vancomycin recruited a broader condition-specific response. Despite differences in scale, both TRNs retained a conserved hierarchical, hub-centered organization, with global regulators bridging multiple neighborhoods and a peripheral ncRNA layer contributing target-level specificity within the transcription-factor scaffold.

A key outcome is the explicit placement of ncRNAs within the antibiotic-stress regulatory hierarchy. We prioritized two inducible *cis*-antisense ncRNAs, ncRNA3340 and ncRNA3683, which are conserved across diverse clinical isolates, display dual-site pairing potential with their *cis* targets, and show energetic profiles consistent with strong *cis* and weaker but favorable *trans* interactions. Expression profiling further supported distinct module behaviors, including coordinated induction for the ncRNA3340-centered set and an inverse ncRNA3683–*cis* target relationship consistent with a putative negative association.

Overall, our integrative framework links network rewiring to ncRNA-centered control during exposure to clinically relevant cell-wall–active antibiotics. These results expand the regulatory map of *E. faecalis* under antibiotic stress and provide prioritized ncRNA putative candidates and target sets for downstream functional analysis of *cis*/*trans* mechanisms and their contribution to antibiotic-adaptive phenotypes.

## Data Availability

The datasets presented in this study can be found in online repositories. The names of the repository/repositories and accession number(s) can be found in the article/Supplementary Material.

## Supplementary material

SUPPLEMENTARY FIGURE S1 Antibiotic titration growth curves used to define non-lethal exposure conditions. Growth kinetics of *Enterococcus faecalis* measured as OD_600nm_ over 480 min under increasing concentrations of (left) vancomycin (Van; 0.31–5 μg/mL) and (right) ceftriaxone (Cef; 125–2,000 μg/mL), compared to the basal condition. Curves show mean values with error bars. Statistical significance relative to basal is indicated (ns = not significant; * = significant).

SUPPLEMENTARY FIGURE S2 Sequence variability profiles of antibiotic-responsive ncRNAs inferred from k-mer entropy. Shannon entropy (bits) computed across k-mer positions along the aligned sequences of (top) ncRNA3340 and (bottom) ncRNA3683. Peaks denote regions of increased sequence variability across the dataset, whereas low-entropy segments indicate higher conservation. Colored intervals highlight variable blocks selected for downstream analyses (e.g., motif/seed-region exploration), and grey traces indicate the remaining positions.

SUPPLEMENTARY TABLE S1 Comprehensive list of nodes included in the transcriptional regulatory network (TRN) constructed for *Enterococcus faecalis* under vancomycin and ceftriaxone exposure.
