# Supplementary figures and images for: Transcriptional regulatory network analysis identifies conserved cis-antisense ncRNAs in the vancomycin and ceftriaxone stress response of Enterococcus faecalis
Source: Front Mol Biosci. 2026 Jun 9;13:1798522. doi: 10.3389/fmolb.2026.1798522 (PMC13286765; doi:10.3389/fmolb.2026.1798522)

## Vancomycin

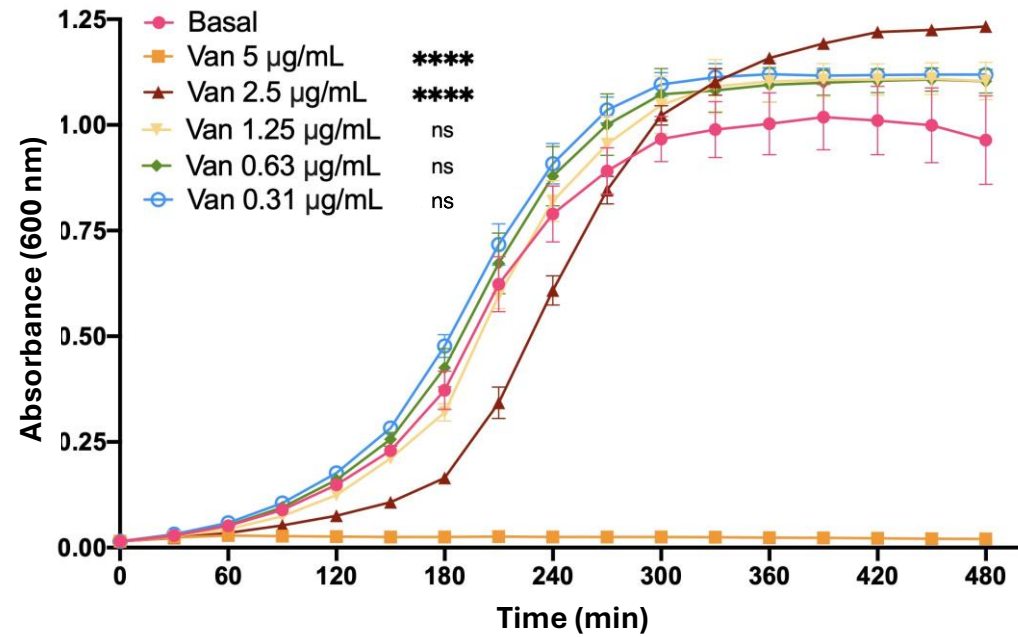

## Ceftriaxone

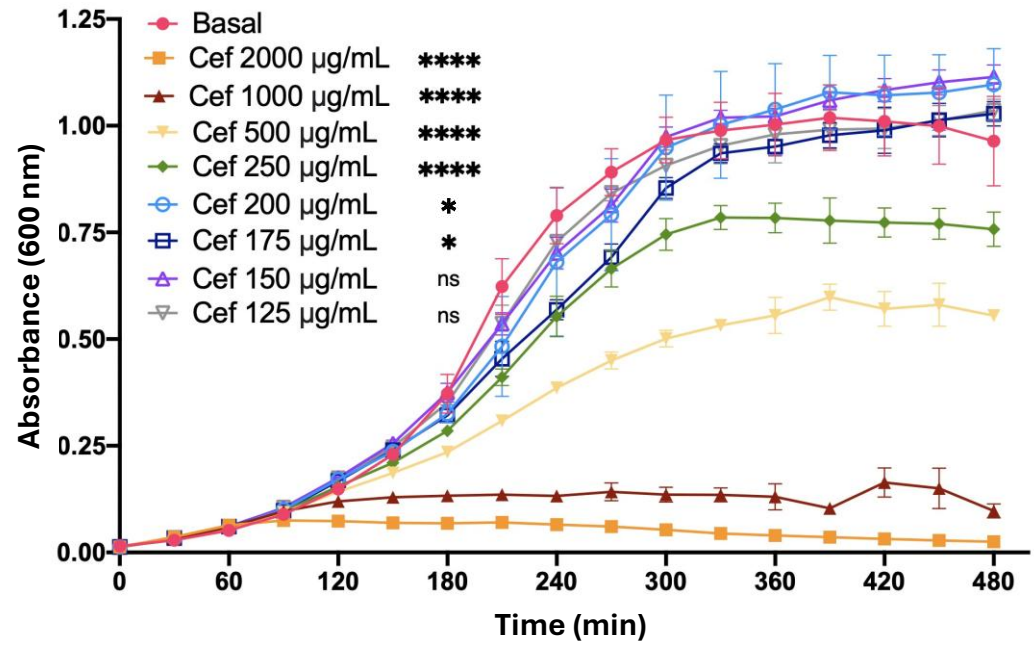

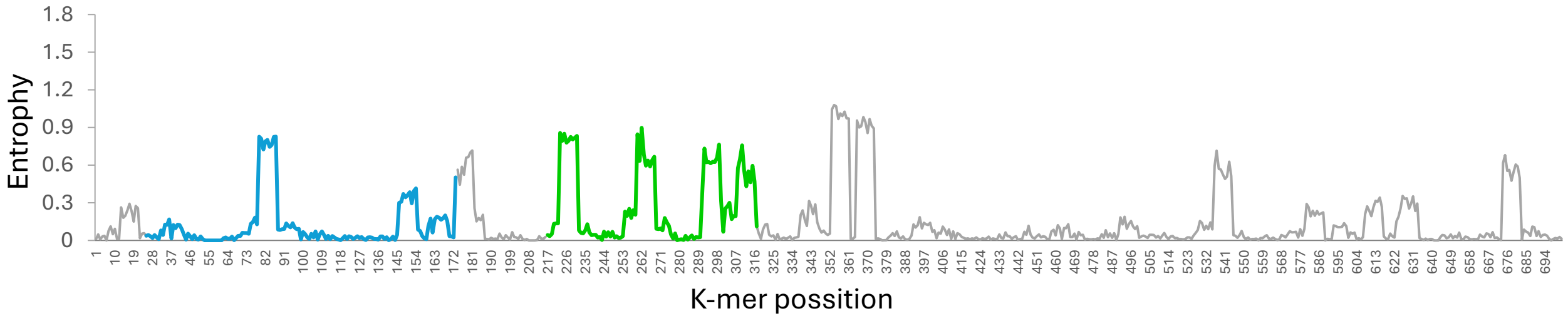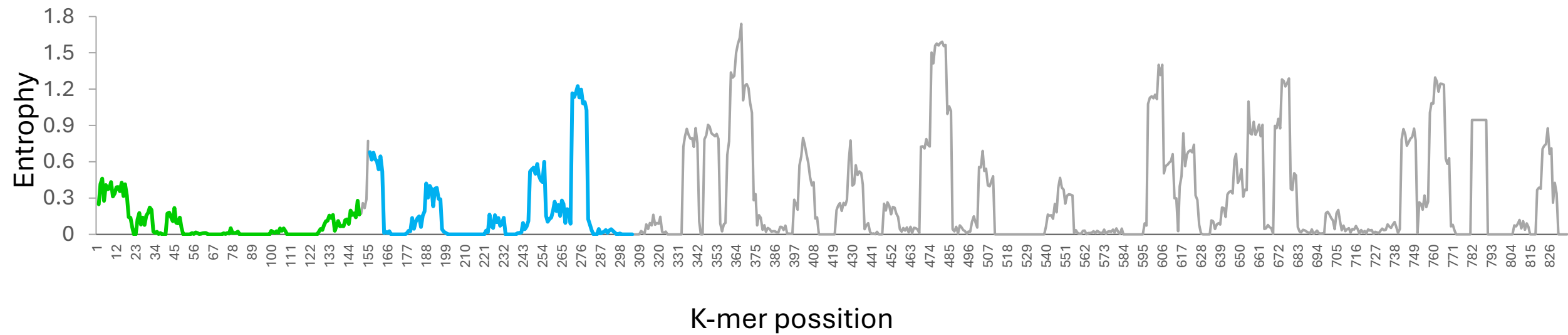

Supplementary Figure 2

Supplement: Supplementary file 2 [file Image1.pdf]
